# Antiphospholipid Antibodies in Lupus Nephritis

**DOI:** 10.1371/journal.pone.0158076

**Published:** 2016-06-23

**Authors:** Ioannis Parodis, Laurent Arnaud, Jakob Gerhardsson, Agneta Zickert, Birgitta Sundelin, Vivianne Malmström, Elisabet Svenungsson, Iva Gunnarsson

**Affiliations:** 1 Department of Medicine, Rheumatology Unit, Karolinska Institutet, Karolinska University Hospital, Stockholm, Sweden; 2 Department of Oncology-Pathology, Karolinska Institutet, Karolinska University Hospital, Stockholm, Sweden; Instituto Nacional de Ciencias Medicas y Nutricion Salvador Zubiran, MEXICO

## Abstract

Lupus nephritis (LN) is a major manifestation of systemic lupus erythematosus (SLE). It remains unclear whether antiphospholipid antibodies (aPL) alter the course of LN. We thus investigated the impact of aPL on short-term and long-term renal outcomes in patients with LN. We assessed levels of aPL cross-sectionally in SLE patients diagnosed with (n = 204) or without (n = 294) LN, and prospectively in 64 patients with active biopsy-proven LN (52 proliferative, 12 membranous), before and after induction treatment (short-term outcomes). Long-term renal outcome in the prospective LN cohort was determined by the estimated glomerular filtration rate (eGFR) and the Chronic Kidney Disease (CKD) stage, after a median follow-up of 11.3 years (range: 3.3–18.8). Cross-sectional analysis revealed no association between LN and IgG/IgM anticardiolipin or anti-β_2_-glycoprotein I antibodies, or lupus anticoagulant. Both aPL positivity and levels were similar in patients with active LN and non-renal SLE. Following induction treatment for LN, serum IgG/IgM aPL levels decreased in responders (p<0.005 for all), but not in non-responders. Both at active LN and post-treatment, patients with IgG, but not IgM, aPL had higher creatinine levels compared with patients without IgG aPL. Neither aPL positivity nor levels were associated with changes in eGFR from either baseline or post-treatment through long-term follow-up. Moreover, aPL positivity and levels both at baseline and post-treatment were similar in patients with a CKD stage ≥3 versus 1–2 at the last follow-up. In conclusion, neither aPL positivity nor levels were found to be associated with the occurrence of LN in SLE patients. However, IgG aPL positivity in LN patients was associated with a short-term impairment of the renal function while no effect on long-term renal outcome was observed. Furthermore, IgG and IgM aPL levels decreased following induction treatment only in responders, indicating that aPL levels are affected by immunosuppressive drugs in a response-dependent manner.

## Introduction

Antiphospholipid antibodies (aPL) constitute a heterogeneous family of antibodies against phospholipids or phospholipid-binding proteins. They may occur in association with autoimmune diseases, transiently in association with infections, and sometimes in the general population. Presence of aPL is associated with enhanced risk of thrombotic manifestations in the arterial, venous and capillary circulation, as well as with pregnancy complications [1–3]. A fraction of individuals with aPL develop the antiphospholipid syndrome (APS) while many remain asymptomatic [4, 5]. APS may appear as an isolated primary syndrome, or as a secondary condition to an underlying disease, systemic lupus erythematosus (SLE) being the most common [6].

Coexistence of aPL along with intrarenal vascular lesions such as thrombotic microangiopathy (TMA), fibrous intimal hyperplasia and focal cortical atrophy constitute a condition called aPL-associated nephropathy (APLN) [1]. Histological findings consistent with APLN were previously described as APS nephropathy (APSN) [7, 8], and studies have also demonstrated that APSN may appear in a limited fraction of SLE patients without aPL [9, 10].

Vascular changes consistent with APLN may be present in renal biopsies from patients with lupus nephritis (LN) [8, 10–12], and have been shown to be associated with the development of end-stage renal disease (ESRD) [10]. Previous studies of the impact of aPL on renal outcomes in LN have demonstrated conflicting results [13–20], and the role of aPL in LN patients without APLN is not thoroughly investigated.

We investigated the occurrence of aPL in patients with LN compared with non-renal SLE patients. Furthermore, we prospectively studied aPL positivity and aPL levels before and after induction treatment and at long-term follow-up in patients with active biopsy-proven LN without concomitant APLN.

## Materials and Methods

### Study design

Since 1995, patients with SLE from the Karolinska University Hospital, Stockholm, Sweden have been enrolled in the Karolinska SLE cohort. The first 498 patients, enrolled between 1995 and 2014, were included in the cross-sectional part of this study. All patients were investigated with regard to aPL at the time of enrolment. Additionally, 64 patients from the Karolinska LN cohort, enrolled between 1996 and 2011 on the occasion of a biopsy-proven active LN without concomitant APLN, were included in the prospective part of the present study. In patients from this cohort, repeated renal biopsies were performed after completion of induction therapy (median time: 7.7 months; range: 5.0–15.6) [21, 22], and aPL levels were measured both at baseline and post-treatment. In order to assess long-term renal outcomes, these patients were followed longitudinally for a median time of 11.3 years (range: 3.3–18.8), counting from the occasion of the first renal biopsy.

All patients fulfilled the 1982 revised criteria [23], as well as the Systemic Lupus International Collaborating Clinics criteria [24], for classification of SLE. Written informed consent was obtained prior to enrolment from all adult individuals participating in the study, and also from the next of kin, caretakers, or guardians on behalf of the minors or children enrolled. The study protocol was reviewed and approved by the regional ethics review board at Karolinska Institutet, Stockholm, Sweden.

### Surveillance methods and definitions

Renal biopsies were evaluated using light, immunofluorescence and electron microscopy. The International Society of Nephrology/Renal Pathology Society (ISN/RPS) 2003 classification of LN [25] was used to classify the patients into LN subsets. Histopathological renal activity and damage were estimated using the Activity Index (AI) and Chronicity Index (CI) [26], respectively.

Global disease activity was assessed using the SLE Disease Activity Index 2000 (SLEDAI-2K) [27]. Urinary status was evaluated by urine test strips and urinary sediment. Proteinuria was estimated by the 24-hour urine albumin excretion (g/day). Renal function was assessed by plasma creatinine concentration (μmol/L) and by the estimated Glomerular Filtration Rate (eGFR), as determined by the Modification of Diet in Renal Disease (MDRD) Study equation [28].

Clinical responders to induction treatment for LN were required to meet three conditions, in line with the American College of Rheumatology response criteria for renal disease in SLE clinical trials [29]: (i) at least 50% reduction in proteinuria to levels ≤2 g/day, (ii) normal eGFR or, if abnormal at baseline, improved by ≥25%, and (iii) an inactive urinary sediment (≤5 red blood cells/high power field, ≤5 white blood cells/high power field and no cellular casts). Cases not meeting these criteria were considered non-responders.

In the prospective LN cohort, the long-term renal outcome was assessed by the last eGFR and the last chronic kidney disease (CKD) stage, as defined by the updated guidelines of the Kidney Disease Outcomes Quality Initiative by the National Kidney Foundation [30–32].

### Determination of autoantibody and immunoglobulin levels

Serum was collected and stored at –80°C on the occasion of enrolment from patients in the cross-sectional part of the study, and both at baseline and post-treatment from patients in the prospective LN cohort. Serum levels of IgG and IgM anticardiolipin antibodies (aCL) and anti-β_2_-glycoprotein I antibodies (anti-β_2_-GPI) (positive values ≥20 U/mL), as well as antibodies to double-stranded DNA (anti-dsDNA; positive values ≥10 IU/mL), were determined by multiplex immunoassays (BioPlex® 2200 System, Bio-Rad Laboratories, Inc., Hercules, California, USA) in all patients for both the cross-sectional (n = 498) and the prospective (n = 64) part of the study. Presence or absence of lupus anticoagulant (LA) was determined by dilute Russell's viper venom time, followed by a confirmatory test. Total immunoglobulin levels were measured by nephelometry.

### Statistics

Data are presented as medians or means (range), or counts (percentage). Associations between current or previous LN and the presence of IgG or IgM aPL, LA, anti-dsDNA and concomitant APS were assessed in the cross-sectional part of the study using logistic regression, and are presented as odds ratios (OR) and their 95% confidence intervals (CI). For comparisons between related samples, the paired samples *t*-test was used for normally distributed variables, and the non-parametric Wilcoxon signed-rank test was used for non-normally distributed samples. Comparisons between independent samples were made using the Student's *t*-test for normally distributed data, and the Mann-Whitney U test for variables with non-normal distributions. Comparisons of proportions between groups were performed using the Pearson Chi-square or the Fisher's exact test. Correlations were performed using the Pearson product-moment correlation coefficient for normally distributed data, and the Spearman’s rank correlation coefficient for non-normally distributed samples. Data from the assessment of autoantibody levels were bounded by the detection limits of the assays. Values under the lower detection limit were set to half the lower limit value, and values over the upper detection limit were set to twice the upper limit value.

To investigate the role of aPL in long-term renal outcomes, as well as in renal activity, renal damage, and global disease activity in the prospective LN cohort, linear mixed models for repeated measures were used. Separate models were built for each outcome of interest (AI, CI, eGFR, proteinuria, and SLEDAI-2K). Each one of these outcomes was separately included as the dependent variable in a linear mixed model, with LN patient visits as repeated and fixed effects, aPL levels as a covariate, and patients as a random effect. For the long-term renal outcome, the model was adjusted for the total observation time in years.

All tests were bilateral and p-values <0.05 were considered statistically significant. In cases of multiple comparisons, the Bonferroni correction was applied. The statistical analyses were performed with the IBM SPSS Statistics 23 software (IBM Corp., Armonk, New York, USA).

## Results

### Patient characteristics and assessment of aPL

Patients from the Karolinska SLE cohort included in the cross-sectional analysis were classified as patients with (n = 204) or without (n = 294) current or previous LN. The prospective LN cohort comprised 64 patients. Patient characteristics for both cohorts are presented in Table 1.

**Table 1:** Patient characteristics.

	SLE cohort: cross-sectional analysis		Prospective LN cohort
	Renal SLE (n = 204)	Non-renal SLE (n = 294)	Baseline (n = 64)
***Sex***			
*Female; n (%)*	162 (79.4%)	266 (90.5%)	55 (86%)
*Male; n (%)*	42 (20.6%)	28 (9.5%)	9 (14%)
***Age*** *(years); M (R)*	42.1 (18.6–81.8)	49.7 (17.3–84.2)	31.7 (18.8–60.7)
***SLE disease duration*** *(years); M (R)*	11.3 (0.0–39.9)	8.0 (0.0–58.2)	3.7 (0.0–35.6)
***Ethnicity***			
*Caucasian; n (%)*	186 (91.2%)	275 (93.5%)	56 (87.5%)
*Asian; n (%)*	7 (3.4%)	7 (2.4%)	3 (4.7%)
*Hispanic; n (%)*	6 (2.9%)	5 (1.7%)	3 (4.7%)
*African; n (%)*	5 (2.5%)	7 (2.4%)	2 (3.1%)
***APS****; n (%)*	40 (22.9%; n = 175)	33 (13.0%; n = 253)	5 (8.1%; n = 62)
***LA ever****; n (%)*	45 (25.9%; n = 174)	53 (21.7%; n = 244)	18 (29.0%; n = 62)
***SLEDAI-2K****; M (R)*	4.0 (0–28)	2.0 (0–20)	16 (6–28)
***Induction treatment***			
*Intravenous cyclophosphamide; n (%)*	-	-	45 (70.3%)
*Mycophenolate mofetil; n (%)*	-	-	11 (17.2%)
*Rituximab; n (%)*	-	-	7 (10.9%)
*Azathioprine; n (%)*	-	-	1 (1.6%)
***Duration of treatment*** *(months); M (R)*	-	-	7.7 (5.0–15.6)

Characteristics of SLE patients with (n = 204) and without (n = 294) current or previous LN in the cross-sectional analysis, and patients in the prospective LN cohort (n = 64).

SLE: systemic lupus erythematosus; LN: lupus nephritis; APS: antiphospholipid syndrome; LA: lupus anticoagulant; SLEDAI-2K: Systemic Lupus Erythematosus Disease Activity Index 2000; M: median; R: range.

In the prospective LN cohort, 52 cases were classified as proliferative LN (ISN/RPS class III/IV±V), and 12 cases as membranous LN (ISN/RPS class V), according to the baseline renal biopsies. None of these patients had a concomitant diagnosis of APLN. Results from evaluation of the renal biopsies, SLEDAI-2K scores, anti-dsDNA levels, total IgG and IgM levels, proteinuria, creatinine values and eGFR are presented in Table 2. No patient was diagnosed with renal artery or vein thrombosis, either concurrently with or prior to LN. Of 63 patients in whom data were available, 7 (11.1%) had a diagnosis of and treatment for diabetes, and 34 (54%) had a diagnosis of and treatment for hypertension. Proportions of patients with aPL and serum aPL levels in the different subgroups are presented in Table 3.

**Table 2:** Comparisons between baseline and post-treatment outcomes.

	Active LN	Treated LN	P-value
***Prednisone equivalent*** *(mg/day); M (R)*	8.8 (0–60); n = 64	10.0 (0–50); n = 64	0.61
***24-h U-albumin*** *(g/day); M (R)*	1.5 (0.04–8.4); n = 63	0.3 (0–4.8); n = 64	**<0.001** ↓
***P-creatinine*** *(μmol/L); M (R)*	81 (46–284); n = 64	76 (40–306); n = 64	**0.009** ↓
***eGFR*** *(mL/min/1*.*73 m*^*2*^*); M (R)*	75 (17–138); n = 64	81 (20–140); n = 64	**0.043** ↑
***ISN/RPS class***			
*I; II (+V); n*	0; 0	1; 15 (1)	-
*III A (+V); III A/C (+V); III C (+V); n*	10 (3); 5 (2); 0	0; 9 (1); 8 (2)	-
*IV S A (+V); IV S A/C (+V); IV S C (+V); n*	4; 3 (1); 0	0; 0; 0	-
*IV G A (+V); IV G A/C (+V); IV G C (+V); n*	9 (3); 11 (1); 0	2; 5 (1); 2	-
*V; n*	12	15	-
*Glomerular vasculitis; n*	0	1	-
***Activity Index****; M (R)*	5 (0–13); n = 64	2 (0–12); n = 63	**<0.001** ↓
***Chronicity Index****; M (R)*	1 (0–6); n = 64	2 (0–8); n = 63	**<0.001** ↑
***SLEDAI-2K****; M (R)*	16 (6–28); n = 64	4 (0–23); n = 64	**<0.001** ↓
***IgG aCL*** *(IU/mL); M (IQR)*	2.0 (0.8–7.5); n = 64	0.8 (0.8–1.9); n = 64	**<0.001** ↓
***IgM aCL*** *(IU/mL); M (IQR)*	0.8 (0.3–2.9); n = 64	0.7 (0.2–2.4); n = 64	**<0.001** ↓
***IgG anti-β***_***2***_***-GPI*** *(U/mL); M (IQR)*	2.0 (0.7–12.0); n = 64	0.7 (0.7–2.7); n = 64	**<0.001** ↓
***IgM anti-β***_***2***_***-GPI*** *(U/mL); M (IQR)*	1.0 (0.4–4.5); n = 64	0.8 (0.2–3.2); n = 64	**<0.001** ↓
***Serum anti-dsDNA*** *(IU/mL); M (IQR)*	110.0 (27.0–600.0); n = 63	20.0 (10.5–71.5); n = 61	**<0.001** ↓
***Total IgG*** *(g/L); M (R)*	15.1 (3.2–25.6); n = 41	10.7 (4.8–29.8); n = 32	**0.01** ↓
***Total IgM*** *(g/L); M (R)*	0.96 (0.05–3.90); n = 40	0.90 (0.04–2.40); n = 32	**0.04** ↓
***IgG aCL/total IgG****; M (R)*	0.2 (0.03–32.0); n = 32	0.08 (0.03–52.5); n = 32	**0.01** ↓
***IgM aCL/total IgM****; M (R)*	1.4 (0.07–24.4); n = 32	0.9 (0.2–40.6); n = 32	0.67
***IgG anti-β***_***2***_***-GPI/total IgG****; M (R)*	0.1 (0.03–32.0); n = 32	0.08 (0.02–52.5); n = 32	**0.02** ↓
***IgM anti-β***_***2***_***-GPI/total IgM****; M (R)*	1.4 (0.1–39.2); n = 32	1.3 (0.2–51.8); n = 32	0.55

Baseline and post-treatment outcomes in the prospective LN cohort. Statistically significant p-values are in bold. Upward arrows (↑) signify significant increases. Downward arrows (↓) signify significant decreases.

LN: lupus nephritis; ISN/RPS: International Society of Nephrology/Renal Pathology Society; SLEDAI-2K: Systemic Lupus Erythematosus Disease Activity Index 2000; anti-dsDNA: antibodies to double-stranded DNA; aCL: antiocardiolipin antibodies; anti-β_2_-GPI: anti-β_2_-glycoprotein I antibodies; (I)U: (international) units; M: median; R: range; IQR: interquartile range.

**Table 3:** Antiphospholipid antibody positivity and levels.

	**Antiphospholipid antibody positivity**; n (%)				**P-value**		
	**Non-renal SLE**	**Renal SLE**	**Active LN**	**Treated LN**	**Active vs. treated LN**	**Active LN vs. non-renal SLE**	**Treated LN vs. non-renal SLE**
	n = 294	n = 204	n = 64	n = 64			
***IgG aCL***	55 (18.7%)	45 (22.1%)	8 (12.5%)	6 (9.4%)	0.32	0.24	0.07
***IgM aCL***	22 (7.5%)	12 (5.9%)	6 (9.4%)	1 (1.6%)	**0.03** ↓	0.61	0.08
***IgG anti-β***_***2***_***-GPI***	57 (19.4%)	49 (24.0%)	9 (14.1%)	6 (9.4%)	0.18	0.32	0.06
***IgM anti-β***_***2***_***-GPI***	23 (7.8%)	13 (6.4%)	6 (9.4%)	2 (3.1%)	**0.046** ↓	0.68	0.18
	**Antiphospholipid antibody levels**; M (IQR)				**P-value**		
	**Non-renal SLE**	**Renal SLE**	**Active LN**	**Treated LN**	**Active vs. treated LN**	**Active LN vs. non-renal SLE**	**Treated LN vs. non-renal SLE**
	n = 294	n = 204	n = 64	n = 64			
***IgG aCL***	0.8 (0.8–7.9)	1.8 (1.0–11.0)	2.0 (0.8–7.5)	0.8 (0.8–1.9)	**<0.001** ↓	0.45	**<0.001**
***IgM aCL***	1.0 (0.6–4.0)	1.0 (0.4–3.0)	0.8 (0.3–2.9)	0.7 (0.2–2.4)	**<0.001** ↓	0.07	**0.001**
***IgG anti-β***_***2***_***-GPI***	0.7 (0.7–9.0)	2.0 (1.0–16.4)	2.0 (0.7–12.0)	0.7 (0.7–2.7)	**<0.001** ↓	0.51	**0.001**
***IgM anti-β***_***2***_***-GPI***	1.1 (0.6–4.1)	1.0 (0.5–3.0)	1.0 (0.4–4.5)	0.8 (0.2–3.2)	**<0.001** ↓	0.22	**0.01**

Counts and proportions of patients with aPL and serum aPL levels in the cross-sectional analysis of SLE patients with (n = 204) and without current or previous LN (n = 294), and in the prospective cohort of biopsy-proven LN (n = 64) before and after completion of induction treatment, as well as comparisons between groups. The units for aCL are IU/mL, and for anti-β_2_-GPI U/mL. The lower limits of the assay were 1.6 IU/mL for IgG aCL, 1.4 U/mL for IgG anti-β_2_-GPI, and 0.2 (I)U/mL for IgM aCL and IgM anti-β_2_-GPI. The upper limit of the assay was 160 (I)U/mL for all aPL. Values <20 (I)U/mL were considered negative. Statistically significant p-values are in bold. Downward arrows (↓) signify significant decreases.

SLE: systemic lupus erythematosus; LN: lupus nephritis; aPL: antiphospholipid antibodies; aCL: anticardiolipin antibodies; anti-β_2_-GPI: anti-β_2_-glycoprotein I antibodies; (I)U: (international) units; M: median; IQR: interquartile range.

### Associations between aPL and LN

In the cross-sectional analysis (n = 498), we found no association between positivity for IgG aCL (OR: 1.23 [95% CI: 0.79–1.91]), IgM aCL (OR: 0.77 [95% CI: 0.37–1.60]), IgG anti-β_2_-GPI (OR: 1.31 [95% CI: 0.85–2.02]), or IgM anti-β_2_-GPI (OR: 0.80 [95% CI: 0.40–1.62]) at the time of enrolment and current or previous LN. Moreover, there was no association between LA positivity at any time prior to enrolment and LN (OR: 1.26 [95% CI: 0.80–1.98]). Further, both aPL positivity and serum levels of aPL were similar in patients with active LN and patients with non-renal SLE (Table 3). In contrast, in the cross-sectional analysis we found that definite diagnosis of APS [1] was associated with current or previous LN (OR: 1.98 [95% CI: 1.19–3.28]; p = 0.009), and, as expected, anti-dsDNA positivity was also associated with current or previous LN (OR: 2.38 [95% CI 1.64–3.64]; p<0.001).

### Associations between aPL and short-term renal outcomes in LN

**Fig 1:**
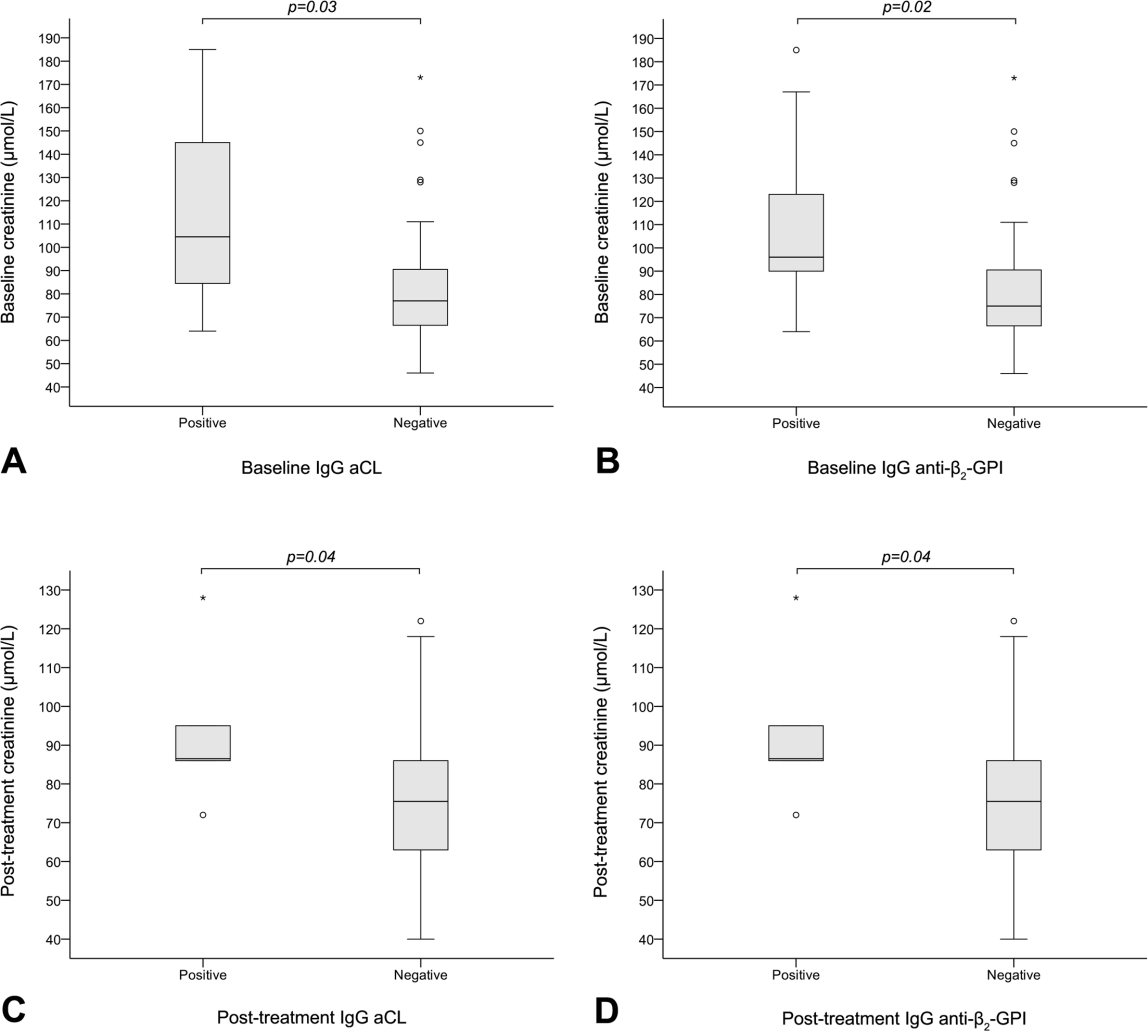
Creatinine levels (μmol/L) in LN patients with and without IgG aPL. At baseline, creatinine levels were higher in LN patients with (n = 8) versus without (n = 56) IgG aCL (A; median: 104.5 μmol/L, range: 64–185, versus 77.0 μmol/L, range: 46–284; p = 0.03). Consistently, creatinine levels were higher in LN patients with (n = 9) versus without (n = 55) IgG anti-β_2_-GPI (B; median: 94.0 μmol/L, range: 64–18, versus 75.0 μmol/L, range: 46–284; p = 0.02). Similar findings were observed post-treatment, with higher creatinine levels in LN patients with (n = 6) versus without (n = 58) IgG aCL (C; median: 86.5 μmol/L, range: 72–128, versus 75.5 μmol/L, range: 40–306; p = 0.04), as well as with (n = 6) versus without (n = 58) IgG anti-β_2_-GPI (D; median: 86.5 μmol/L, range: 72–128, versus 75.5 μmol/L, range: 40–306; p = 0.04). Bounds of the boxes denote the 25^th^ and 75^th^ percentiles (IQR). Lines in the boxes denote the 50^th^ percentile (median). Whiskers denote the range. Circles (out values, 1.5–3 IQRs further from the closest box bound) and stars (far out or extreme values, ≥3 IQRs further from the closest box bound) denote outliers. Some extreme values do not appear in the figure due to scaling. LN: lupus nephritis; aCL: anticardiolipin antibodies; anti-β_2_-GPI: anti-β_2_-glycoprotein I antibodies.

In the prospective LN cohort, creatinine levels at baseline were higher in LN patients with versus without IgG aCL (p = 0.03) and anti-β_2_-GPI (p = 0.02), but were similar in LN patients with and without IgM aCL (p = NS) or anti-β_2_-GPI (p = NS). Similar findings were observed post-treatment (Fig 1).

In contrast, no correlation was found between serum aPL levels and Activity or Chronicity Index scores in renal biopsies, SLEDAI-2K, 24-h U-albumin, anti-dsDNA levels, or age, either at baseline or post-treatment (p = NS for all).

Following induction treatment, we observed decreased proportions of patients with IgM aCL (p = 0.03) and IgM anti-β_2_-GPI (p = 0.046), while proportions of patients with IgG aPL remained unchanged (Table 3). When we investigated serum levels of aPL, both IgG and IgM isotypes decreased following treatment (p<0.001 for all; Table 3). In order to investigate whether the reductions in aPL levels were dependent on the induction treatment regimen, we stratified the patients of the prospective LN cohort into patients treated with cyclophosphamide or rituximab (CYC/RTX, n = 52) and patients treated with mycophenolate mofetil (MMF, n = 11). Levels of IgG/IgM aCL and anti-β_2_-GPI showed decreases in both treatment groups (Table 4).

**Table 4:** Comparisons with regard to the induction treatment regimen.

Prospective LN cohort	Active LN	Treated LN	P-value
***IgG aCL***			
*CYC/RTX; n = 52*	2.0 (0.8–8.2)	0.8 (0.8–2.0)	**<0.001** ↓
*MMF; n = 11*	1.9 (0.8–4.8)	0.8 (0.8–0.8)	**0.03** ↓
***IgM aCL***			
*CYC/RTX; n = 52*	0.9 (0.3–4.0)	0.8 (0.3–2.8)	**0.001** ↓
*MMF; n = 11*	0.6 (0.2–1.9)	0.4 (0.1–1.2)	**0.007** ↓
***IgG anti-β***_***2***_***-GPI***			
*CYC/RTX; n = 52*	2.0 (0.7–14.0)	0.7 (0.7–3.7)	**<0.001** ↓
*MMF; n = 11*	2.4 (0.7–5.2)	0.7 (0.7–1.4)	**0.03** ↓
***IgM anti-β***_***2***_***-GPI***			
*CYC/RTX; n = 52*	1.1 (0.4–5.0)	1.0 (0.3–3.3)	**0.002** ↓
*MMF; n = 11*	0.9 (0.3–2.4)	0.6 (0.2–1.3)	**0.007** ↓

Comparisons between baseline and post-treatment aPL levels in the prospective LN cohort (n = 64), with regard to the induction treatment regimen. Data are presented as medians (IQR). Levels of aCL are in IU/mL. Levels of anti-β_2_-GPI are in U/mL. Downward arrows (↓) signify significant decreases.

aPL: antiphospholipid antibodies; LN: lupus nephritis; aCL: anticardiolipin antibodies; anti-β_2_-GPI: anti-β_2_-glycoprotein I antibodies; CYC: cyclophosphamide; RTX: rituximab; MMF: mycophenolate mofetil; (I)U: (international) units; M: median; IQR: interquartile range.

In order to clarify whether these reductions were due to decreases in the total immunoglobulin levels following treatment, we compared the ratios of aPL levels to total immunoglobulin levels before and after treatment. Although total IgG and IgM levels decreased following treatment (p = 0.02 and p = 0.01, respectively; Table 2), we observed that the ratios of IgG aPL to total IgG also decreased for both aCL (p = 0.01) and anti-β_2_-GPI (p = 0.02), while ratios of IgM aCL to total IgM (p = 0.67) and IgM anti-β_2_-GPI to total IgM remained stable (p = 0.55; Table 2).

Numbers of clinical responders and non-responders to induction treatment are reported in Table 5. Baseline aPL levels did not differ between patients who responded to the treatment and patients who did not show clinical improvements (p = NS for all comparisons; Table 5). We observed reductions in serum levels of both IgG (p<0.001) and IgM (p = 0.002) aCL, as well as IgG (p<0.001) and IgM (p = 0.003) anti-β_2_-GPI, in responders, but not in non-responding patients (Table 5). In contrast, anti-dsDNA levels decreased in both responding (p<0.001) and non-responding (p = 0.02) LN patients.

**Table 5:** Comparisons with regard to clinical response to induction treatment.

Prospective LN cohort	Active LN	Treated LN	P-value	
			Active vs. treated LN	Baseline aPL levels in R vs. NR
***IgG aCL***				
*Responders*	2.6 (0.8–8.2)	0.8 (0.8–2.0)	**<0.001** ↓	
*Non-responders*	0.8 (0.8–1.9)	0.8 (0.8–0.8)	0.07	
				0.07
***IgM aCL***				
*Responders*	0.8 (0.2–3.9)	0.6 (0.1–2.6)	**0.002** ↓	
*Non-responders*	0.9 (0.4–2.9)	1.0 (0.2–2.1)	0.03	
				0.55
***IgG anti-β***_***2***_***-GPI***				
*Responders*	2.6 (0.7–13.0)	0.7 (0.7–3.2)	**<0.001** ↓	
*Non-responders*	0.7 (0.7–3.0)	0.7 (0.7–1.5)	0.03	
				0.18
***IgM anti-β***_***2***_***-GPI***				
*Responders*	1.0 (0.3–4.6)	0.7 (0.2–3.2)	**0.003** ↓	
*Non-responders*	0.9 (0.5–4.5)	1.3 (0.3–3.1)	0.03	
				0.65

Comparisons between baseline and post-treatment aPL levels in the prospective LN cohort (n = 64), with regard to clinical response to induction treatment, and comparisons of baseline aPL levels in clinical responders (R; n = 48) versus non-responders (NR; n = 16). Data are presented as medians (IQR). Levels of aCL are in IU/mL. Levels of anti-β_2_-GPI are in U/mL. P-values in bold remained statistically significant after Bonferroni correction. Downward arrows (↓) signify significant decreases after Bonferroni correction.

aPL: antiphospholipid antibodies; LN: lupus nephritis; aCL: anticardiolipin antibodies; anti-β_2_-GPI: anti-β_2_-glycoprotein I antibodies; R: responders; NR: non-responders; (I)U: (international) units; M: median; IQR: interquartile range.

### Long-term renal outcomes

In the prospective LN cohort, the long-term follow-up median eGFR was 80 mL/min/1.73 m^2^ (range: 17–149), and patients were stratified into CKD stages (stage 1, n = 22; stage 2, n = 26; stage 3, n = 12; stage 4, n = 3). No patient had developed ESRD (CKD stage 5). Six patients died during follow-up. In these cases, long-term renal outcomes were evaluated based on the last available blood tests. One patient was lost to follow-up. Long-term follow-up eGFR did not differ from eGFR at either active LN (p = 0.79) or post-treatment (p = 0.21).

Neither baseline nor post-treatment aPL levels correlated with the long-term follow-up eGFR (p = NS), or were associated with long-term changes in eGFR (p = NS). Long-term eGFR did not differ between aPL positive and negative LN patients either at baseline or post-treatment (p = NS for all). Consistently, neither aPL positivity nor serum levels of aPL, either at baseline or post-treatment, differed between LN patients with CKD stage 1–2 and patients with CKD stage ≥3 at the last follow-up (p = NS).

## Discussion

We investigated the role of aPL in short-term and long-term renal outcomes in patients with biopsy-proven LN without concomitant APLN. We observed higher creatinine levels in LN patients with IgG aPL, both at active disease and after treatment. We also demonstrated that serum levels of aPL decreased following induction treatment in responders, but not in non-responders. Of note, IgG aPL levels decreased independently of decreasing total IgG levels. However, we found no association between aPL and the long-term renal outcome.

Results from previous investigations of the significance of aPL in LN have been conflicting. A previous study demonstrated higher aCL levels in LN compared with non-renal SLE patients [33] while recently IgM anti-β_2_-GPI were found to be protective against LN [15]. In our cross-sectional analysis of SLE patients, we found no association between aPL positivity and LN. Moreover, neither aPL positivity nor serum levels of aPL differed between patients with active LN and SLE patients without current or previous LN. Although the apparent discrepancy between our results and those from other studies may partly be due to different study designs and different methods used to assess aPL levels, our data suggest that aPL *per se* are not associated with the occurrence of LN. Surprisingly, we found an association between definitely diagnosed APS and LN. However, it is known that aPL positive individuals do not always develop symptoms [4, 5], and patients with APS are likely carriers of more pathogenic aPL.

Previous studies found no association between aPL and histopathological activity or chronicity features in LN [14, 34]. In line with these findings, we found no correlation between aPL and AI or CI scores in either baseline or post-treatment renal biopsies in our prospective LN cohort. In contrast, we found higher creatinine levels in LN patients with IgG aPL compared with patients without, both at active LN and after induction treatment. A previous study demonstrated higher creatinine levels in patients with anti-neutrophil cytoplasmic autoantibody (ANCA)-associated small vessel vasculitis and IgG anti-plasminogen antibodies, a pivotal component of the fibrinolytic system, compared with patients without such antibodies [35]. Given the expected cross-reactivity between IgG anti-plasminogen and IgG anti-β_2_-GPI antibodies [36], this observation may be considered consistent with ours. Taken together, our findings suggest that IgG aPL might contribute to an impaired renal function during a LN flare despite the absence of APLN, and raise the hypothesis that aPL may have a pathogenic role in the kidney, resulting in renal function deterioration. Immunohistochemistry studies of aPL expression in renal tissue from LN patients might shed light on the mechanisms behind this and contribute to further understanding.

Surprisingly, aPL levels decreased in LN patients who responded to induction treatment, including patients with aPL levels below the cut-off value for positivity, but remained stable in non-responding patients, in contrast to anti-dsDNA levels which decreased regardless of treatment outcomes. Of note, baseline aPL levels did not differ between responders and non-responding patients. This suggests that the decreases of aPL levels were unlikely due to a general effect of immunosuppression on immunoglobulin levels, which was also supported by the differences between baseline and post-treatment ratios of IgG aPL to total IgG levels. The discrepancy in the behaviour of aPL in responding versus non-responding patients suggests that aPL, especially IgG aPL, may reflect and possibly contribute to a more severe LN phenotype. However, it is important to underline that aPL levels below the cut-off value for positivity have a questionable clinical significance, being the reason why we investigated the behaviour of both aPL levels and aPL positivity following treatment for nephritis.

Previous studies have consistently demonstrated associations of aCL [9, 10], anti-β_2_-GPI [12], and LA [8, 12] with APSN, as well as between APSN and the development of ESRD [10]. However, investigations of the impact of aPL on renal outcomes in LN have been conflicting, demonstrating associations of aPL with renal function deterioration in some studies [13], no association with long-term renal outcomes in others [14], and even a protective role of IgM anti-β_2_-GPI against renal damage in a recent report [15]. Here, we were able to confirm an association of aPL with renal function impairment during a LN flare in a short-term perspective, but we found no protective role of IgM anti-β_2_-GPI against renal activity or damage.

Further, we found no association between either the presence or serum levels of aPL and additional renal function deterioration in the long term. This might indicate that aPL *per se* do not contribute to the long-term renal outcome in patients with LN in the absence of APLN. Supportive of this hypothesis was also a recent study of 349 SLE patients, which demonstrated that aPL did not predict irreversible renal damage [37], as assessed by the Systemic Lupus International Collaborating Clinics (SLICC)/American College of Rheumatology (ACR) Damage Index (SDI) [38]. However, firm conclusions about the impact of aPL on the long-term renal outcome cannot be drawn from our study due to the sample size and the limited proportion of patients with aPL in the prospective LN cohort.

A recent study showed that the renal vascular expression of annexin A2, a phospholipid-binding protein [39] with an important role in the pathogenesis of APS [40–43] and LN [44], did not differ between patients with LN and patients with other kidney diseases. Interestingly, annexin A2 expression was more intense in patients with vascular lesions consistent with APLN [45]. Deeper surveys of aPL expression in renal tissue from patients with LN, as well as from patients with APLN, are needed in order to clarify their pathogenic role and hopefully contribute to better and more specific treatment approaches in selected cases.

## Conclusions

In this study, we found no association of either aPL positivity or levels with the occurrence of LN. In patients with LN, IgG aPL may contribute to a short-term impairment of the renal function, but no effect on the long-term renal outcome was observed. Furthermore, reductions of IgG and IgM aPL levels were noted in LN patients who responded to induction treatment, but not in non-responders, indicating that aPL levels are affected by immunosuppressive drugs in a response-dependent manner. Our findings merit further investigation of aPL in LN, in order to determine their expression and functional role on a tissue level.
